# Risk Stratification in Cardiovascular Medicine for Prognostic Assessment and Therapeutic Decision‐Making: From Atrial Fibrillation to the Broader Disease Spectrum

**DOI:** 10.1002/joa3.70435

**Published:** 2026-07-29

**Authors:** Yuichi Saito, Kazuya Tateishi, Ken Kato, Hideki Kitahara, Yoshio Kobayashi

**Affiliations:** ^1^ Department of Cardiovascular Medicine Chiba University Hospital Chiba Japan

**Keywords:** cardiovascular disease, diagnosis, prognosis, risk score

## Abstract

Risk stratification using validated scoring systems is fundamental to contemporary cardiovascular medicine, underpinning diagnosis, treatment selection, and prognostic assessment. This review provides a comprehensive overview of the major risk scores used across a broad spectrum of cardiovascular diseases, such as atrial fibrillation, acute coronary syndrome, and others, highlighting their development, clinical applications, and evolving roles in guideline‐directed care. Importantly, predictive performance alone does not guarantee clinical benefit. We therefore examine evidence from randomized implementation trials evaluating whether risk score‐guided management improves clinical outcomes and discuss the future role of artificial intelligence in reshaping cardiovascular risk assessment by integrating dynamic, individualized prediction with actionable clinical decision support.

## Graphical abstract

Risk stratification using validated scoring systems plays a significant role in contemporary cardiovascular medicine not only for prognostic assessment but also for treatment optimization, resource allocation, and shared decision‐making. Beyond accurate prognostication, whether the use of a risk score can aid clinical decision‐making and improve patient outcomes is relevant.

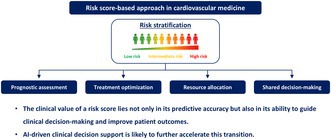

## 1 Introduction

**FIGURE 1:**
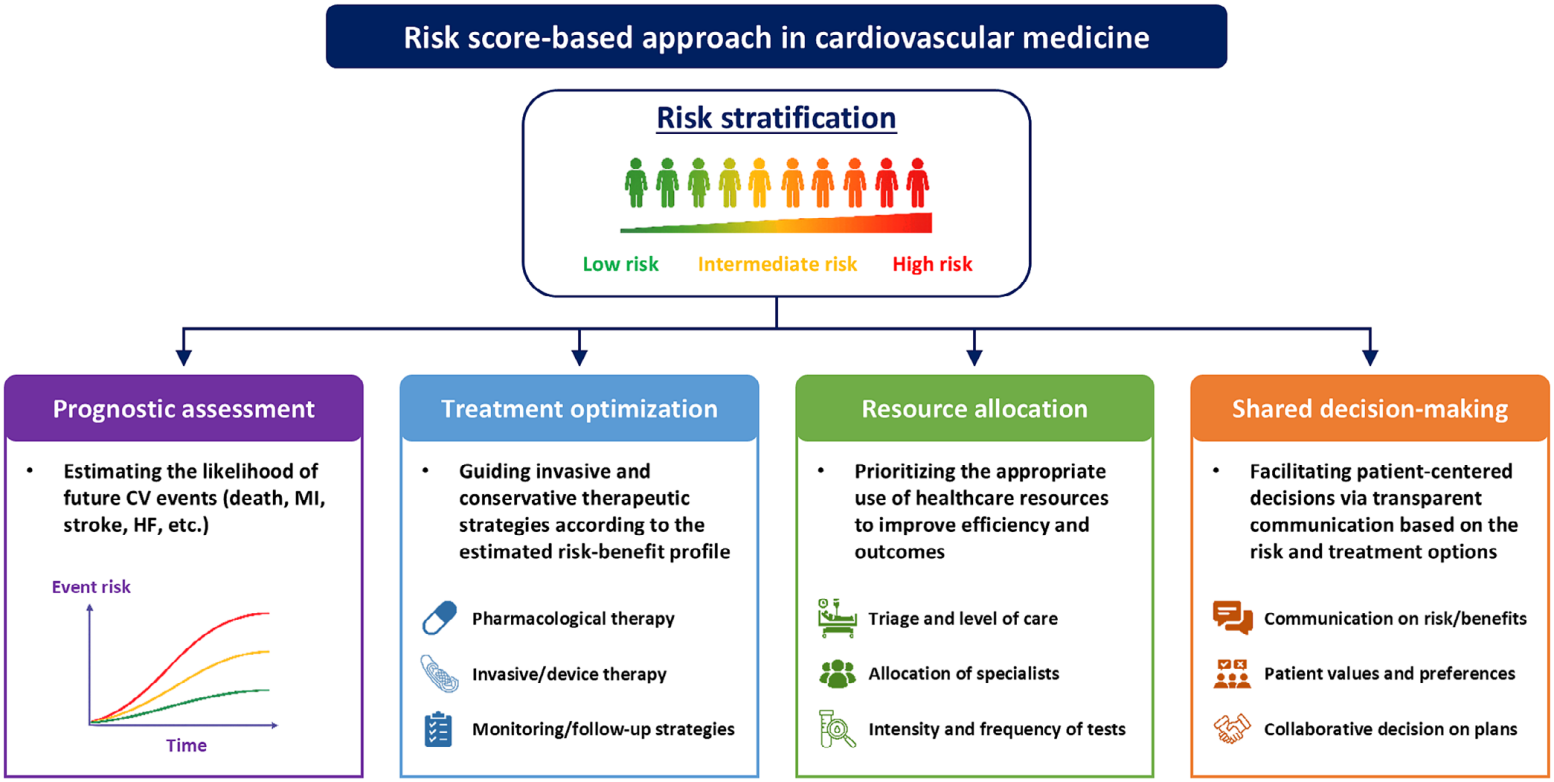
Roles of risk score‐based approach in cardiovascular medicine. CV, cardiovascular; HF, heart failure; MI, myocardial infarction.

Cardiovascular diseases remain the leading cause of global mortality and morbidity, accounting for an estimated 18.6 million deaths annually and an enormous burden on healthcare systems worldwide [1, 2]. In cardiovascular medicine, risk stratification using validated scoring systems plays a central role in contemporary clinical practice. The Framingham Risk Score is one of the earliest risk models in a primary prevention cohort for predicting future ischemic heart disease, developed based on decades of epidemiological follow‐up data from the landmark Framingham Heart Study [3]. In primary prevention settings, the PREVENT equations in the United States (US) and the SCORE2 risk prediction algorithms in European countries are the guideline‐recommended strategies for predicting atherosclerotic cardiovascular disease in current clinical practice [4, 5]. In secondary prevention settings, numerous risk scores, such as the CHADS_2_ score in atrial fibrillation (AF) and the GRACE risk score in acute coronary syndrome (ACS) [6, 7], have been proposed. Given the substantial heterogeneity among patients in real‐world clinical practice, accurate estimation of future cardiovascular risk is essential for individualized management. Such risk stratification has important clinical implications not only for prognostic assessment and the optimization of treatment strategies, but also for the efficient allocation of healthcare resources and the effective communication of risk with patients and their significant others. In the era of personalized medicine, risk‐guided management has become increasingly important for balancing treatment benefits and potential harms, improving clinical outcomes, and supporting patient‐centered care (Figure 1). In the present review article, we provide a comprehensive, domain‐by‐domain overview of the major risk scores used across the spectrum of cardiovascular disease (Table 1). Additionally, we focus on the emerging evidence from randomized implementation trials that have tested whether risk score‐guided care improves clinical outcomes and also discuss how artificial intelligence (AI) technologies will reshape the landscape of cardiovascular risk assessment.

**TABLE 1:** Major risk scores in cardiovascular medicine across clinical domains.

Risk score (year)	Region of origin	Components	Predicted outcomes
Atrial fibrillation			
CHADS_2_ (2001)	US	CHF, hypertension, age, diabetes, prior stroke/TIA	Stroke
CHA_2_DS_2_‐VASc (2010)	Europe	CHF, hypertension, age, diabetes, stroke/TIA/thromboembolism, vascular disease, female sex	Thromboembolism
HELT‐E_2_S_2_ (2021)	Japan	Age, hypertension, BMI, type of AF, prior stroke	Ischemic stroke
HAS‐BLED (2010)	Europe	Hypertension, abnormal renal/liver function, stroke, bleeding history, labile INR, elderly, drugs/alcohol	Major bleeding events
ATRIA (2011)	US	Anemia, severe renal disease, age, prior bleeding, hypertension	Major bleeding events
ORBIT (2015)	US	Age, reduced hemoglobin/anemia, bleeding history, renal impairment, antiplatelet therapy	Major bleeding events
Acute coronary syndromes and chest pain			
TIMI (2000)	US	Age, coronary risk factors, known coronary stenosis, ST deviation, anginal episodes, aspirin, cardiac markers	Mortality and coronary events
GRACE (2006)	International	Age, heart rate, systolic BP, serum creatinine, Killip class, cardiac arrest, ST deviation, cardiac biomarkers	In‐hospital and 6‐month mortality
HEART Pathway (2015)	US	History, ECG, age, risk factors, serial troponin	Mortality and coronary events
Heart failure			
MAGGIC (2013)	International	LVEF, age, systolic BP, BMI, creatinine, NYHA class, sex, smoking, diabetes, COPD, HF duration, β‐blocker, ACE‐i/ARB	Mortality in chronic HF
EHMRG30‐ST (2019)	Canada	Age, EMS transport, systolic BP, heart rate, oxygen saturation, potassium, creatinine, troponin, active cancer, prior metolazone, ST depression	30‐day mortality in acute HF
Venous thromboembolism			
Wells score (2000)	Canada	Clinical DVT signs, heart rate, immobilization or surgery, prior PE or DVT, hemoptysis, malignancy, PE most likely diagnosis	Pre‐test probability of PE
PESI (2005)	US	Age, sex, cancer, HF, chronic lung disease, pulse rate, systolic BP, RR, body temperature, altered mental status, oxygen saturation	30‐day mortality after PE
sPESI (2010)	Spain	Age, cancer, chronic cardiopulmonary disease, pulse rate, systolic BP, oxygen saturation	30‐day mortality after PE
Cardiac (cardiovascular) surgery			
STS Score (2009)	US	Approximately 30–40 variables of patient characteristics and procedure‐related factors	Operative mortality and complications
Euro SCORE II (2012)	International	18 variables of patient characteristics and procedure‐related factors	Operative mortality
Japan SCORE (2008)	Japan	Approximately 20–30 variables of patient characteristics and procedure‐related factors	Operative mortality and complications
Primary prevention			
SCORE2 (2021)	Europe	Age, smoking, systolic BP, total/HDL cholesterol, region, sex	10‐year CV events (age 40–69 years)
SCORE2‐OP (2021)	Europe	Age, smoking, systolic BP, total/HDL cholesterol, region, sex	10‐year CV events in age ≥ 70 years
PREVENT (2024)	US	Age, sex, total/HDL cholesterol, systolic BP, antihypertensives, diabetes, smoking, BMI, eGFR (optional: HbA1c, UACR, SDI)	10‐ and 30‐year ASCVD and HF events
Hisayama Score (2022)	Japan	Age, sex, systolic BP, HDL and LDL cholesterol, proteinuria, smoking, regular exercise	10‐year ASCVD events

Abbreviations: ACE‐i, angiotensin‐converting enzyme inhibitor; AF, atrial fibrillation; ARB, angiotensin receptor II blocker; ASCVD, atherosclerotic cardiovascular disease; BMI, body mass index; BP, blood pressure; CHF, congestive heart failure; COPD, chronic obstructive pulmonary disease; CV, cardiovascular; DVT, deep venous thrombosis; ECG, electrocardiography; eGFR, estimated glomerular filtration rate; EMS, emergency medical services; HbA1c, glycated hemoglobin; HDL, high‐density lipoprotein; HF, heart failure; INR, international normalized ratio; LDL, low‐density lipoprotein; LVEF, left ventricular ejection fraction; NYHA, New York Heart Association; PE, pulmonary embolism; RR, respiratory rate; SDI, Social Deprivation Index; TIA, transient ischemic attack; UACR, urinary albumin‐to‐creatinine ratio; US, United States.

## 2 Key Examples of Risk Scoring Systems in Cardiovascular Medicine

### 2.1 Atrial Fibrillation

AF is the most common sustained cardiac arrhythmia and is associated with thromboembolic events, heart failure (HF), and mortality [8, 9]. In the Framingham Study, the presence of AF was associated with a 4‐ to 5‐fold increased risk of ischemic stroke [10]. Thus, the development of risk stratification tools for stroke has been central to the management of AF, as the decision to initiate oral anticoagulation (OAC) requires balancing stroke prevention against bleeding risk. AF represents one of the best examples of risk‐stratified management in cardiovascular medicine. Stroke and bleeding risks are routinely assessed using validated risk scores, and treatment strategies, particularly decisions regarding OAC, are guided by these assessments in contemporary clinical practice and international guidelines. The original CHADS_2_ score was introduced in 2001 to predict the risk of thromboembolic events, integrating 5 clinical variables—congestive HF, hypertension, age ≥ 75 years, diabetes, and prior stroke or transient ischemic attack (Table 1); [6]. The CHADS_2_ score was incorporated into the 2006 US guidelines for AF as a risk stratification tool [11], but subsequent analyses revealed an important limitation that it classified a large proportion of patients into an intermediate‐risk category, providing insufficient guidance for treatment decisions in this substantial group [12]. The CHA_2_DS_2_‐VASc score was proposed in 2010 to address these limitations by incorporating additional stroke risk factors, including vascular disease, age 65–74 years, and female sex category [12]. The validation studies have shown the superior discriminative ability of the CHA_2_DS_2_‐VASc score over the CHADS_2_ score [13]. and current guidelines recommend the former for thromboembolic risk stratification, although the traditional CHA_2_DS_2_‐VASc score was further replaced by the CHA_2_DS_2_‐VA score in the 2024 European guidelines for AF, which excluded female sex as a risk modifier [14]. As scientific evidence accumulates, risk scores undergo continuous refinement and adaptation. The evolution from CHADS_2_ to CHA_2_DS_2_‐VASc and, more recently, CHA_2_DS_2_‐VA represents one of the most prominent examples of the successful integration of risk stratification tools into everyday clinical practice. However, the diagnostic ability of the CHA_2_DS_2_‐VASc score for stroke or systemic embolism is limited, with a reported C‐statistic of 0.675 in a meta‐analysis of 8 studies [15], and whether the CHADS_2_ scores‐based anticoagulation approach can improve outcomes as compared to no risk score‐based assessment remains untested. Additionally, although the CHA_2_DS_2_‐VASc scores are the most widely used tools for thromboembolic risk stratification, concerns have been raised regarding their generalizability across different racial and ethnic populations [16]. In Japan, the domestically proposed HELT‐E_2_S_2_ score is currently recommended for risk stratification (Class IIa) [17].

The decision to use OAC involves balancing stroke prevention against bleeding risk. The HAS‐BLED score, incorporating hypertension, abnormal renal/liver function, stroke, bleeding history, labile international normalized ratio of prothrombin time, elderly, and drugs/alcohol, was introduced in 2010 to provide a validated assessment of major bleeding risk in patients with AF [18]. A high HAS‐BLED score should not preclude the use of OAC but should instead be used to identify and manage modifiable bleeding risk factors [14]. Similar to the series of CHADS_2_ scores, the discriminative ability of the HAS‐BLED score for major bleeding events was limited, with a reported C‐statistic of 0.63 in a meta‐analysis [19]. Other bleeding risk tools include the ATRIA and ORBIT scores, although the HAS‐BLED remains the most widely endorsed score in guidelines [8, 14].

### 2.2 Acute Coronary Syndrome and Chest Pain in the Emergency Department

Chest pain is one of the most common reasons for emergency department (ED) visits, accounting for approximately 5%–10% of all presentations [20]. To rule out serious conditions such as ACS and guide subsequent management, objective risk assessment tools are essential in clinical practice. The GRACE risk score represents the most comprehensively validated prognostic tool for patients with ACS, which was derived from multinational registry data, integrating 8 clinical variables available at presentation (Table 1); [7]. Several observational studies support the prognostic ability of the GRACE risk score in patients with non‐ST‐elevation ACS (NSTEACS) [21, 22, 23], and the score is recommended as a Class I indication in the international guidelines for ACS [24, 25]. Patients with (suspected) NSTEACS and a high GRACE score (> 140) are recommended for an early invasive strategy within 24 h [24]. Similar to the CHADS_2_ scores, the GRACE risk score has evolved from the original version to versions 2.0 and 3.0 [26, 27]. Of note, the GRACE risk score is one of the few risk prediction tools that have been evaluated in randomized trials of a risk‐guided strategy [28, 29]. The TIMI risk score is another well‐validated model in patients with NSTEACS (Table 1); [30]. While demonstrating strong specificity for major adverse cardiac events in high‐risk patients (e.g., TIMI risk score ≥ 6), its sensitivity for excluding adverse events in low‐risk patients is limited compared with the GRACE risk score [31].

Unlike the GRACE and TIMI risk scores, which were developed for patients with confirmed ACS, the HEART score, consisting of 5 components–patient‐reported medical history, electrocardiographic changes, age, atherosclerotic risk factors, and cardiac troponin–was designed for the broader population of undifferentiated chest pain patients presenting to the ED [32]. A meta‐analysis of 25 studies comprising 25 266 patients confirmed the strong performance of the low HEART score with a pooled sensitivity of 96% and negative predictive value of 99% for short‐term major adverse cardiac events [33]. The subsequent HEART Pathway is a decision aid designed to identify patients visiting the ED with acute chest pain for early discharge, in which the HEART score was combined with serial troponin measurements at 0 and 3 h [34]. In a randomized trial, the HEART Pathway decreased objective cardiac testing by 12.1% (56.7% vs. 68.8%, *p* = 0.048), reduced median length of stay by 12 h (9.9 vs. 21.9 h, *p* = 0.013), and increased early discharges by 21.3% (39.7% vs. 18.4%, *p* < 0.001), compared with usual care [34]. The HEART Pathway, therefore, serves as an exemplary model for cardiovascular risk stratification [35], demonstrating that the ultimate value of a risk score lies in the capacity to guide clinical decision‐making and improve healthcare delivery when implemented in routine practice.

### 2.3 Heart Failure

HF represents a major and growing global health burden [36], and accurate risk stratification is essential for guiding clinical decision‐making, optimizing resource allocation, and improving patient outcomes. The MAGGIC risk score, derived from individual patient data across 30 studies (*n* = 39 372), was developed to predict 1‐ and 3‐year all‐cause mortality in patients with chronic HF with either reduced or preserved ejection fraction [37]. The score sums integer points from 13 routinely available variables: left ventricular ejection fraction, age, systolic blood pressure, body mass index, serum creatinine, New York Heart Association class, male sex, current smoking, diabetes, chronic obstructive pulmonary disease, HF duration (≥ 18 months), and absence of *β*‐blocker and angiotensin‐converting enzyme inhibitor or angiotensin‐receptor blocker therapy. It showed modest prognostic ability with a C‐statistic of 0.71, confirmed by external validation [37]. Despite being developed more than a decade ago, the MAGGIC risk score remains a well‐established and widely used tool for prognostic risk stratification in patients with chronic HF.

In addition to predicting long‐term prognosis, optimizing the management of patients with HF in acute care settings, particularly in the ED, remains a key challenge in contemporary clinical practice. Previous studies have shown that when ED disposition decisions for acute HF were based on clinical judgment alone, some high‐risk patients were prematurely discharged, while some low‐risk patients were unnecessarily admitted [38]. The EHMRG and its subsequent iteration, EHMRG30‐ST, were developed to predict 7‐day and 30‐day mortality in patients presenting to the emergency department with acute HF [39]. These scores incorporate variables available at presentation, including age, transport by emergency medical services, heart rate, systolic blood pressure, oxygen saturation, potassium, creatinine, cardiac troponin, active cancer, prior use of metolazone, and ST‐segment depression on 12‐lead electrocardiography. The landmark COACH trial demonstrated that a hospital‐based strategy incorporating the EHMRG30‐ST risk tool for clinical decision support–combined with a rapid outpatient follow‐up program for discharged patients–can improve outcomes in patients with acute HF visiting the ED, compared with usual care, as described below [40]. The development, validation, and subsequent implementation of the EHMRG30‐ST represent an important example of implementation science in cardiovascular medicine.

### 2.4 Venous Thromboembolism

The Wells score was developed to categorize patients into low, moderate, and high pre‐test probability groups for deep vein thrombosis and pulmonary embolism [41, 42]. A notable limitation of the Wells score is the inclusion of a subjective criterion as a strong component, namely, whether pulmonary embolism is considered the most likely diagnosis, which may introduce interobserver variability [42]. The fact that a risk score incorporating such a subjective clinical judgment has been widely adopted may reflect the inherent challenges associated with the diagnosis of pulmonary embolism.

Contemporary diagnosis of pulmonary embolism follows a sequential, Bayesian framework—as codified in the European guidelines—in which clinical pretest probability is assessed first, followed by D‐dimer testing, and finally contrast‐enhanced computed tomography [43]. The principal aim of the available scoring systems is to safely reduce unnecessary imaging tests. These tools can be grouped into three functional layers. The first comprises pretest probability scores, including the Wells score, the revised Geneva score, and the recent 4‐level Clinical Pretest Probability Score (4PEPS) [44]. The second layer is represented by the Pulmonary Embolism Rule‐out Criteria (PERC), an 8‐item model to exclude pulmonary embolism without D‐dimer or imaging tests in patients who are deemed to be very low risk [45]. The third layer includes probability‐adjusted D‐dimer strategies for venous thromboembolism [46, 47]. Comparative data indicate that these adjusted strategies substantially reduce imaging utilization but entail a modest trade‐off in sensitivity relative to fixed or age‐adjusted cutoffs, and their performance varies across special populations. Accordingly, institutions are best served by adopting a single, internally consistent diagnostic pathway, thereby minimizing both missed diagnoses and D‐dimer or computed tomography overuse. These instruments remain adjuncts to, rather than substitutes for, clinical judgment. For patients with established pulmonary embolism, the Pulmonary Embolism Severity Index (PESI) and its simplified version (sPESI) provide prognostic information to guide management intensity [48, 49, 50].

### 2.5 Cardiovascular Surgery

Cardiac surgery is one of the highest‐risk surgical interventions, and a variety of risk scoring systems have been developed to assess and stratify perioperative risk in this setting. The EuroSCORE, developed from a multinational database across 128 centers in 8 European countries, was one of the first comprehensive surgical risk models to estimate perioperative mortality [51]. The original EuroSCORE, which incorporated patient‐related factors (age, sex, and comorbidities), cardiac factors (ventricular function and recent myocardial infarction), and operation‐related factors (type and setting of surgery), showed good discriminative ability with a C‐statistic of 0.79 [51]. However, as cardiac surgery techniques and perioperative care improved over time, the original EuroSCORE tended to overestimate mortality, particularly in high‐risk patients [52]. Thus, the revised EuroSCORE II was introduced in 2012, which incorporated updated risk factors, replaced the simplistic additive model with a logistic framework, and demonstrated improved calibration while maintaining discrimination (C‐statistic 0.81) [53]. The EuroSCORE II remains one of the most widely used risk stratification tools in cardiac surgery worldwide, while the EuroSCORE 3 has recently been developed to recalibrate risk prediction in contemporary cardiac surgical populations.

In the US, the Society of Thoracic Surgeons (STS) risk score has become a cornerstone of risk assessment in cardiac surgery. The model was developed and validated using data from the STS National Database, one of the largest clinical registries of cardiac surgical procedures worldwide [54]. In contrast to the EuroSCORE, which provides a single model for all cardiac surgical procedures, the STS score offers procedure‐specific models for isolated coronary artery bypass grafting, isolated valve surgery, and combined procedures. The STS risk score has evolved through continuous recalibration and periodic updating based on data from the STS database rather than through discrete generational versions. Meta‐analyses showed that the STS score and EuroSCORE II performed comparably in terms of discriminative ability, although there may be regional variation [55]. Given racial differences, the JapanSCORE was developed from the national database to estimate the risk of standard cardiovascular surgery, specifically for the Japanese population [56]. In the current clinical practice worldwide, both the EuroSCORE II and STS score play a central role in Heart Team decision‐making.

### 2.6 Primary Prevention

In primary prevention populations, accurate long‐term estimation of cardiovascular risk is crucial for guiding preventive strategies and optimizing risk factor management. The Framingham Risk Score, one of the most influential epidemiological milestones in cardiovascular medicine, was the first widely adopted multivariable risk prediction tool for primary prevention of coronary heart disease [57]. Incorporating age, sex, total and high‐density lipoprotein cholesterol levels, systolic blood pressure, treatment for hypertension, smoking status, and diabetes, the Framingham model estimated the 10‐year risk of coronary disease events. Subsequently, the Pooled Cohort Equations were developed in 2013 in the US, using diverse and updated cohort data to predict 10‐year risk of atherosclerotic cardiovascular events, including not only coronary but also cerebrovascular outcomes [58]. Most recently, the PREVENT equations were introduced in 2024 in the US, derived from individual‐level data of more than 6 million participants across 25 datasets [59]. The PREVENT equations offer several important advances: it is sex‐specific but race‐independent; it incorporates renal function as a predictor; it adjusts for competing risk of non‐cardiovascular death; and it predicts not only atherosclerotic disease but also HF events [59]. Of note, the PREVENT provides both 10‐year and 30‐year risk estimates for US adults aged 30–79 years. The equations have demonstrated accurate prediction across diverse populations [60], and the recent guidelines strongly recommend the equations [5].

In Europe, the academic society transitioned from the original SCORE to SCORE2 in 2021, which estimates the 10‐year risk of both fatal and non‐fatal cardiovascular events in individuals aged 40–69 without prior cardiovascular disease or diabetes [4]. The SCORE2 employs sex‐specific, competing risk–adjusted models incorporating age, smoking status, systolic blood pressure, and cholesterol levels. A critical innovation is the calibration to 4 regional risk levels across European countries (low, moderate, high, and very high), acknowledging that baseline cardiovascular risk varies substantially by region [4]. The SCORE2‐OP extends these predictions to individuals aged ≥ 70 years [61]. The European guidelines recommend the SCORE2 in cardiovascular risk assessment in primary prevention cohorts for guiding preventive strategies [62]. In Japan, Hisayama Score was developed to estimate the risk of 10‐year atherosclerotic cardiovascular disease [63]. Nonetheless, it remains uncertain whether risk score‐guided treatment strategies in primary prevention settings translate into more efficient and effective prevention of future cardiovascular disease.

## 3 From Prediction to Implementation

The preceding section catalogues a comprehensive portfolio of validated risk scores across cardiovascular medicine. Although widely incorporated into clinical guidelines, predictive accuracy alone does not guarantee clinical benefit. Risk models have limited value if they fail to influence therapeutic decisions. Ultimately, the value of a risk score should be judged not by how well it predicts outcomes with external validation, but by whether its use improves clinical decision‐making and patient outcomes. Importantly, several pragmatic randomized trials have evaluated whether risk score‐guided management improves clinical outcomes, thereby providing direct evidence of the clinical utility of selected risk scores (Table 2).

**TABLE 2:** Randomized trials of risk score‐guided management strategies in cardiovascular care.

Risk score	Trial	Population	Design	Sample size	Score‐guided strategy	Key findings
GRACE	AGRIS	ACS	Cluster RCT	2318	GRACE score‐guided invasive strategy and medical therapy	Invasive management higher in experimental arm; no difference in outcomes at 12 months
GRACE	UKGRIS	NSTEACS	Cluster RCT	3050	GRACE score‐guided invasive strategy and medical therapy	No improvement in guideline‐directed management and outcomes at 12 months
EHMRG30‐ST	COACH	Acute HF at ED	Stepped‐wedge cluster RCT	5452	Early discharge in low‐risk patients and hospital admission in high‐risk patients	Lower incidence of death and CV hospitalization at 30 days in the experimental arm
HEART	HEART Pathway	Chest pain at ED	Single‐center RCT	282	HEART Pathway‐guided early discharge or further evaluation	Objective cardiac testing and LOS reduced without MACE in early‐discharge patients
DAPT	PARTHENOPE	PCI	Multicenter RCT	2107	Score‐guided DAPT duration (3, 6, or 24 months) vs. standard 12 months	NACE reduced in the score‐guided group with fewer ischemic events

Abbreviations: ACS, acute coronary syndrome; CV, cardiovascular; DAPT, dual antiplatelet therapy; ED, emergency department; HF, heart failure; LOS, length of stay; MACE, major adverse cardiac events; NACE, net adverse clinical events; NSTEACS, non‐ST‐elevation acute coronary syndrome; PCI, percutaneous coronary intervention; RCT, randomized controlled trial.

In patients with NSTEACS, a GRACE risk score > 140 has been used to identify high‐risk patients for whom an early invasive strategy within 24 h should be considered [24, 25]. This guideline recommendation has been supported mainly by subgroup analyses of randomized trials, such as TIMACS and VERDICT, in which the benefit of earlier invasive evaluation appeared greatest among patients with the GRACE score > 140 [64, 65]. However, trials evaluating routine GRACE score‐guided decision support, including AGRIS and UKGRIS, have not consistently demonstrated improvements in care processes or clinical outcomes. The AGRIS and subsequent UKGRIS represent landmark cluster randomized trials that moved beyond evaluating the predictive performance of the GRACE risk score to testing whether its routine implementation could improve clinical care and patient outcomes [28, 29]. These pragmatic trials addressed the more clinically relevant question of whether risk‐guided management changes practice. The AGRIS randomized Australian hospitals to routine GRACE risk assessment with linked treatment recommendations or usual care. Although the use of early invasive management modestly increased in the intervention arm, it did not significantly improve overall adherence to guideline‐recommended care or reduce death or myocardial infarction, and the trial was terminated early for futility [28]. Similarly, the UKGRIS trial evaluated routine GRACE‐ and CRUSADE‐guided management across 42 hospitals within the UK National Health Service. Despite the successful implementation of a structured risk assessment pathway, no significant improvements were observed in guideline‐directed care, cardiovascular outcomes, quality of life, or length of hospital stay, apart from a modest increase in referral to cardiac rehabilitation [29]. Importantly, these neutral findings should not be interpreted as evidence against the value of risk stratification itself. Instead, they highlight the challenges of translating prognostic information into improved clinical outcomes. Process evaluations from the AGRIS demonstrated that simply providing a validated risk score was insufficient to change clinician behavior or integrate risk assessment into routine workflows. Collectively, the AGRIS and UKGRIS underscore that the clinical utility of a risk score depends on effective implementation strategies that translate risk estimates into actionable clinical decisions.

The HEART Pathway provided another complementary piece of evidence in the context. The randomized trial (*n* = 282) demonstrated that structured risk stratification for undifferentiated chest pain significantly reduced cardiac testing, shortened hospital stays, and increased safe early discharges, as mentioned above [34]. A subsequent study across 3 US sites confirmed these benefits persisted through 1‐year follow‐up [66]. Key to the HEART Pathway's success was that it addressed a well‐documented problem of overutilization—the majority of patients visiting the ED with chest pain (> 80%) do not have ACS, yet the evaluation is costly and prolonged. The risk score identified a clear, actionable subgroup (low‐risk patients) for whom a specific action, early discharge, can be safely achieved.

In contrast to the neutral findings of AGRIS and UKGRIS, the COACH trial demonstrated that risk score‐guided management can improve patient outcomes when coupled with an effective implementation strategy [40]. This stepped‐wedge, cluster‐randomized trial enrolled 5452 patients with acute HF across 10 Canadian hospitals and evaluated a hospital‐wide intervention integrating the EHMRG30‐ST risk score into clinical decision support. Rather than providing risk estimates alone, the intervention linked risk stratification to predefined management pathways, including disposition guidance and rapid outpatient follow‐up for discharged patients. The intervention reduced inappropriate early discharge of high‐risk patients and significantly lowered the composite of all‐cause death or cardiovascular hospitalization within 30 days. The success of COACH likely reflects several key design features. First, the intervention addressed a genuine clinical decision point at the ED where objective risk assessment could meaningfully influence management. Second, the risk score was embedded within routine clinical workflows as an electronic decision support tool. Finally, risk estimates were directly linked to actionable care pathways.

Although numerous risk models have been developed to estimate ischemic and bleeding risk after percutaneous coronary intervention (PCI) [67, 68, 69, 70, 71, 72, 73, 74, 75, 76], evidence that treatment modification based on these predictions improves clinical outcomes has been limited. Another compelling example of risk score‐guided therapeutic decision‐making is the PARTHENOPE trial, which evaluated a personalized dual antiplatelet therapy (DAPT) strategy after PCI [77]. Unlike conventional fixed‐duration DAPT, the intervention tailored treatment duration according to the DAPT score, assigning shorter DAPT to patients at lower ischemic risk and prolonged DAPT to those with higher predicted ischemic benefit. This risk‐guided approach significantly reduced net adverse clinical events compared with the standard 12‐month strategy while maintaining a similar risk of major bleeding. The PARTHENOPE therefore provides randomized evidence that validated risk scores can successfully guide treatment selection rather than simply predict prognosis. Together with the COACH trial, these findings illustrate that risk score‐guided strategies are most effective when risk estimates are directly linked to actionable therapeutic decisions, highlighting the transition of cardiovascular risk scores from prognostic instruments to tools for precision medicine.

## 4 Future Directions With Artificial Intelligence

Although contemporary cardiovascular risk scores have become indispensable in clinical practice, several inherent limitations constrain their effectiveness. Most provide a static assessment at a single time point and cannot capture the dynamic evolution of patient risk. They are typically derived from regression models using a limited number of preselected variables, and thus, it is challenging to model complex nonlinear interactions or integrate high‐dimensional clinical data. Furthermore, calibration may deteriorate over time as populations, diagnostic modalities, and therapeutic strategies evolve. Another practical limitation is that individual scores address isolated clinical questions, whereas real‐world patients often require simultaneous assessment of multiple competing risks. Importantly, implementation studies such as AGRIS and UKGRIS have demonstrated that accurate risk prediction alone does not necessarily translate into improved clinical practice or patient outcomes unless risk estimates are embedded within actionable clinical workflows [28, 29]. AI offers promising solutions to many of these limitations. Unlike conventional risk scores, machine learning models can integrate large‐scale multimodal data–including serial laboratory measurements, electrocardiographic and imaging features, electronic health records, genomics, and others–to generate continuously updated, individualized risk estimates [78, 79]. Recent meta‐analyses have demonstrated superior discrimination of machine learning models compared with traditional risk scores for cardiovascular risk prediction, while emerging AI systems have shown the ability to derive prognostic information directly from clinically available data [80].

Beyond prediction, AI has the potential to bridge the critical gap between risk estimation and therapeutic decision‐making. Rather than calculating multiple independent risk scores, future AI‐driven clinical decision support systems could automatically extract patient information from electronic health records, simultaneously estimate ischemic, bleeding, procedural, and comorbidity risks, and provide evidence‐based, patient‐specific treatment recommendations that evolve as new clinical information becomes available. Such systems would extend the concept demonstrated in the COACH trial by seamlessly linking risk assessment with actionable care pathways [40]. However, several important challenges remain, including model interpretability, external validation, regulatory oversight, algorithmic fairness, and, most importantly, prospective randomized trials demonstrating improvements in patient outcomes. In an era of rapidly evolving cardiovascular diagnosis and treatment [81, 82, 83, 84, 85, 86, 87, 88, 89, 90, 91, 92, 93, 94, 95, 96, 97, 98, 99, 100, 101, 102, 103, 104, 105, 106, 107, 108, 109, 110, 111, 112, 113, 114, 115, 116, 117, 118, 119, 120, 121, 122, 123, 124, 125, 126, 127, 128, 129], the future success of risk prediction will depend not only on increasingly accurate models but also on their effective integration into clinical workflows to support timely and evidence‐based therapeutic decisions.

## 5 Conclusions

Cardiovascular risk scores have transformed risk assessment across the spectrum of cardiovascular medicine. Yet their true clinical value lies not in prediction alone, but in their ability to inform therapeutic decisions and improve patient outcomes. In an era of rapidly evolving cardiovascular care and artificial intelligence, the next frontier is to integrate accurate, dynamic risk prediction with intelligent clinical decision support. Ultimately, success will be measured not by how well we predict risk, but by how effectively we translate risk knowledge into better patient care.

## Funding

The authors have nothing to report.

## Conflicts of Interest

Yuichi Saito has received lecture fees from Daiichi Sankyo, Novartis, and Novo Nordisk. Yoshio Kobayashi has received lecture fees from Amgen, Novartis, Medtronic Japan, and Daiichi Sankyo and research grants from Abbott Medical Japan, Win International, Nipro, Kaneka Medics, and OrbusNeich Medical.

## Data Availability

Data sharing not applicable to this article as no datasets were generated or analysed during the current study.
